# Supplementary material for: Anti-nociceptive action of peripheral mu-opioid receptors by G-beta-gamma protein-mediated inhibition of TRPM3 channels
Source: eLife. 2017 Aug 15;6:e26280. doi: 10.7554/eLife.26280 (PMC5593507; doi:10.7554/eLife.26280)
Supplement: Transparent reporting form [file elife-26280-transrepform.pdf]

1st Floor
24 Hills Road
Cambridge CB2 1JP, UK

P 01223 855340
W elifesciences.org
T @elife

eLife's transparent reporting form

We encourage authors to provide detailed information *within their submission* to facilitate the interpretation and replication of experiments. If you have any questions, please contact us: editorial@elifesciences.org.

Sample-size estimation

- You should state whether an appropriate sample size was computed when the study was being designed
- You should state the statistical method of sample size computation and any required assumptions
- If no explicit power analysis was used, you should describe how you decided what sample (replicate) size (number) to use

Please outline where this information can be found within the submission (e.g., page numbers or figure legends), or explain why this information doesn't apply to your submission:

As detailed in the manuscript, we did not perform a-priori estimation of sample sizes, but followed closely the standards in the field. Further considerations for our choices of sample sizes are detailed in the manuscript.

Replicates

- You should report how often each experiment was performed
- You should include a definition of biological versus technical replication
- The data obtained should be provided and sufficient information should be provided to indicate the number of independent biological and/or technical replicates
- If you encountered any outliers, you should describe how these were handled
- Criteria for exclusion/inclusion of data should be clearly stated
- High-throughput sequence data should be uploaded before submission, with a private link for reviewers provided (these are available from both GEO and ArrayExpress)

Please outline where this information can be found within the submission (e.g., page numbers or figure legends), or explain why this information doesn't apply to your submission:

The procedures and principles that guided our statistical analysis are outlined in the "Methods section" (subsection entitled "*Data analysis, representation and statistical testing*").

eLIFE
1st Floor
24 Hills Road
Cambridge CB2 1JP, UK
P 01223 855340
W elifesciences.org
T @elife

Statistical reporting

- Statistical analysis methods should be described and justified
- Raw data should be presented in figures whenever informative to do so (typically when N per group is less than 10)
- For each experiment, you should identify the statistical tests used, exact values of N, definitions of center, methods of multiple test correction, and dispersion and precision measures (e.g., mean, median, SD, SEM, confidence intervals; and, for the major substantive results, a measure of effect size (e.g., Pearson's r, Cohen's d)
- Report exact p-values wherever possible alongside the summary statistics and 95% confidence intervals. These should be reported for all key questions and not only when the p-value is less than 0.05.

Please outline where this information can be found within the submission (e.g., page numbers or figure legends), or explain why this information doesn't apply to your submission:

(For large datasets, or papers with a very large number of statistical tests, you may upload a single table file with tests, Ns, etc., with reference to page numbers in the manuscript.)

- The statistical methods used are detailed in "Methods" section (subsection entitled "*Data analysis, representation and statistical testing*").
- We show raw data for all small data sets.
- The exact number of "n" and the number of independent experiments used to obtain these data are stated in the figures legends. The statistical tests used are described in the Methods section (see above).
- We used only non-parametric tests (which is stated in detail in the manuscript), but exact p-values were not reported for all tests by the software package that we used (Graphpad Prism, version 3.02 or R). We have compared the results obtained with Graphpad Prism to the results from a more recent software package (Wavemetrics Igor v7), but also this software package does not always give exact p-values (e.g. for Dunn's multiple comparison test), or the large number of samples necessitates the calculation of p-values with approximation techniques, severely limiting their interpretational value. Finally, we have recalculated all statistical tests with parametric tests (Students, ANOVA with Newman-Keuls post-tests).
We found that the non-parametric and parametric tests give similar results, as long as we use $p < 0.05$ as criterion for rejecting H0.

eLife Sciences Publications, Ltd is a limited liability non-profit non-stock corporation incorporated in the State of Delaware, USA, with company number 5030732, and is registered in the UK with company number FC030576 and branch number BR015634 at the address 1st Floor, 24 Hills Road, Cambridge CB2 1JP | August 2014

eLIFE
1st Floor
24 Hills Road
Cambridge CB2 1JP, UK
P 01223 855340
W elifesciences.org
T @elife

Additional data files ("source data")

- We encourage you to upload relevant additional data files, such as numerical data that are represented as a graph in a figure, or as a summary table
- Where provided, these should be in the most useful format, and they can be uploaded as "Source data" files linked to a main figure or table
- Include model definition files including the full list of parameters used
- Include code used for data analysis (e.g., R, MatLab)
- Avoid stating that data files are "available upon request"

Please indicate the figures or tables for which source data files have been provided:

Not applicable, we do not provide a source file

eLife Sciences Publications, Ltd is a limited liability non-profit non-stock corporation incorporated in the State of Delaware, USA, with company number 5030732, and is registered in the UK with company number FC030576 and branch number BR015634 at the address 1st Floor, 24 Hills Road, Cambridge CB2 1JP | August 2014
